# Near-Universal Prevalence of *Pneumocystis* and Associated Increase in Mucus in the Lungs of Infants With Sudden Unexpected Death

**DOI:** 10.1093/cid/cis870

**Published:** 2012-10-16

**Authors:** Sergio L. Vargas, Carolina A. Ponce, Miriam Gallo, Francisco Pérez, J.-Felipe Astorga, Rebeca Bustamante, Magali Chabé, Isabelle Durand-Joly, Pablo Iturra, Robert F. Miller, El Moukthar Aliouat, Eduardo Dei-Cas

**Affiliations:** 1 Programa de Microbiología, Instituto de Ciencias Biomédicas, Facultad de Medicina Universidad de Chile; 2 Departamento de Tanatología, Servicio Médico Legal, Santiago, Chile; 3 Center for Infection and Immunity of Lille, Institut Pasteur de Lille, INSERM U1019, UMR CNRS 8204, Biology and Diversity of Emerging Eukaryotic Pathogens; 4 Department of Parasitology-Mycology, EA4547, Faculty of Pharmacy; 5 Department of Microbiology, Faculty of Medicine, CHRU de Lille, University Lille Nord de France; 6 Research Department of Infection and Population Health, University College London, United Kingdom

**Keywords:** immunocompetent, non-specific immune response, autopsy, MUC5AC, Sudden Infant Death Syndrome (SIDS)

## Abstract

***Background.*** *Pneumocystis* without obvious accompanying pathology is occasionally reported in autopsied infant lungs. Its prevalence and significance are unknown. Interestingly, this mild infection induces a strong activation of mucus secretion–related genes in young immunocompetent rodents that has not been explored in infants. Excess mucus is induced by multiple airway offenders through nonspecific pathways and would explain a cofactor role of *Pneumocystis* in respiratory disease. We undertook characterization of the prevalence of *Pneumocystis* and associated mucus in infant lungs.

***Methods.*** Samples from 128 infants (mean age, 101 days) who died suddenly and unexpectedly in Santiago during 1999–2004 were examined for *Pneumocystis* using nested polymerase chain reaction (nPCR) amplification of the *P. jirovecii* mtLSU ribosomal RNA gene and immunofluorescence microscopy (IF). *Pneumocystis*-negative infants 28 days and older and their age-closest positives were studied for MUC5AC expression and *Pneumocystis* burden by Western blot and quantitative PCR, respectively.

***Results.*** *Pneumocystis* DNA was detected by nPCR in 105 of the 128 infants (82.0%) and *Pneumocystis* organisms were visualized by IF in 99 (94.3%) of the DNA-positive infants. The infection was commonest at 3–4 months with 40 of 41 (97.6%) infants of that age testing positive. MUC5AC was significantly increased in *Pneumocystis-*positive tissue specimens (*P* = .013). Death was unexplained in 113 (88.3%) infants; *Pneumocystis* was detected in 95 (84.0%) of them vs 10 of 15 (66.7%) with explained death (*P* = .28).

***Conclusions.*** A highly focal *Pneumocystis* infection associated to increased mucus expression is almost universally present in the lungs of infants dying unexpectedly in the community regardless of autopsy diagnosis.

## Abstract (precis)

We demonstrate that *Pneumocystis* reaches a >90% prevalence peak at 3–5 months of age and associates with increased mucus (MUC5AC), suggesting airway epithelium stimulation in infants during this age range. Host ability to clear mucus would determine pathogenic expression.

**(See the Editorial Commentary by Eddens and Kolls, on pages 180–1.)**

Most humans experience their first contact with *Pneumocystis* (ie, primary infection) shortly after birth [1–4]. This infection is rarely diagnosed because it is asymptomatic or may present as a mild upper respiratory infection [4–6]. Autopsy reports of *Pneumocystis* in infants have been available for many years [7]. However, characterization of this infection has been hampered by the lack of a microbiological culture method for *Pneumocystis*, by the low sensitivity of any method used to diagnose *Pneumocystis* pneumonia in the immunocompromised to detect the smaller quantities of this fungus in immunocompetent individuals, and because *Pneumocystis* cysts do not stain with the standard hematoxylin-and-eosin stain routinely used in most autopsies. Recent autopsy studies describe the focal (patchy) histological distribution [8–10] and that this infection is more frequent between the ages of 2 and 5 months [4, 5, 9–11]. This age range coincides with the most frequent age for sudden unexpected infant death (SUID) and bronchiolitis [12, 13]. However, the coverage extent of this age overlap and whether it carries any pathogenic significance for *Pneumocystis* are unknown. Increasing evidence shows that *Pneumocystis* induces a potent immune response in young immunocompetent rodents [3, 14–17], including a strong gene activation of ClCa3, a member of the calcium-activated chloride channel family of genes expressed in the goblet airway epithelial cell that relates to mucus secretion [14]. Mucus is produced constitutively by goblet cells and binds virtually all particles that land in the airway epithelium as an essential component of the mucociliary clearance system aimed to clean the airways from inhaled particles. This system comprises secretory and ciliated cells, a periciliary liquid (PCL) layer where the cilia move to impulse the mucus, and the propelled overlaying mucus [18]. The heights of the PCL and of the mucus layers need fine tuning to secure airway patency while maintaining clearance efficiency [18–20]. Excess PCL will raise the floating mucus layer, making it unreachable to cilia for propulsion, and accumulating mucus could occlude narrow, developing, and distal airways [18–21]. Mucus release is an airway defense reaction stimulated through nonspecific pathways by multiple airway offenders [19–21]. A *Pneumocystis*-related increase in mucus would then suggest a cofactor role for *Pneumocystis* in lung disease of the immunocompetent host that is nearly undetectable with current autopsy procedures. Therefore, we undertook this cross-sectional study to describe the prevalence, age distribution, and mucus-associated response to the primary infection by *Pneumocystis* in autopsied infant lungs.

## MATERIALS AND METHODS

### Ethics Review

This study was approved by the Ethics Commissions of the North Metropolitan Area of Health, and of the University of Chile School of Medicine in Santiago.

### Study Population and Data Collection

The Servicio Médico Legal in Santiago is the coroners' office institution for the Metropolitan Area of Chile. A medico-legal autopsy is required for infants who have died in the community in Chile. Infant autopsies performed during calls of a thanatology specialist physician (M.G.) between 1 May 1999 and 6 July 2004 were selected for the study. Inclusion criteria were unexpected death at home, no hospital admission, no immunocompromising conditions, and normal macroscopic examination. The forensic protocol considered clinical history, macroscopic examination and dissection with histological sampling of major organs, plus laboratory tests including toxicology determinations. No bacterial or viral cultures were considered. Medical information including age, date of death, findings including lung histology report, and autopsy diagnoses were collected from the coroner's report prior to *Pneumocystis* analyses. Autopsy diagnoses were categorized for the purpose of this study as (1) unexplained death (no abnormal findings at autopsy, sudden infant death syndrome); (2) unexplained death with autopsy findings whose contributory role to death was uncertain; and (3) explained death, when a definitive cause of death was established. (Groups 1, 2, and 3 would correspond to SUID or sudden unexpected death in infancy [12, 13]).

### Autopsy Samples

The complete right lung was carefully removed, placed in a sterile plastic bag, and transported to the investigatoŕs laboratory in an ice-pack container after obtaining legally required samples using sterile equipment. Each lung was processed at arrival, one at a time; lobes were dissected inside a biosafety cabinet using new sterile equipment as described [22]. The pleura was carefully removed to access untouched tissue using separate sterile equipment. Small samples were obtained from deep lung tissue through 2-cm-deep multiple incisions in the decorticated surface of each lobe. Specimens were cut into small pieces and distributed for nested polymerase chain reaction (nPCR) and microscopy. Lobes were processed and analyzed separately.

### Samples for *Pneumocystis* Categorization

DNA was extracted and purified from a median of 0.172 g (mean, 0.168 g [range, 0.099–0.226 g]) of pulmonary tissue using the QIAamp DNA Mini Kit (Qiagen, Valencia, California) monitoring for cross-contamination [22]. *Pneumocystis jirovecii* DNA was identified by nPCR using human β-globin internal controls [22]. Standard cleaning and sterilization procedures using DNA breaking fluids (DNA Away, VWR Scientific Products) were applied to the biosafety cabinet and hood units between each lung.

Infants were categorized as *Pneumocystis* positive when the *P. jirovecii* DNA–specific 267 bp band was visualized in 1 or more specimens, and as *Pneumocystis* negative if no *P. jirovecii* DNA was documented in the 3 lobes. *Pneumocystis-*negative lobes were analyzed twice, starting from tissue.

### Microscopy Analyses

A median of 0.396 g (mean, 0.399 g [range, 0.319–0.498 g]) of lung tissue was homogenized by magnetic stirrer agitation in sterile phosphate-buffered saline (PBS) pH 7.2 at 4°C for 30 minutes, sterile gauze filtered, centrifuged at 2900*g*, 10 minutes at 4°C, and the pellet was reconstituted in 700 µL of sterile PBS pH 7.2. Five-microliter drops were used for microscopy slides. Forms of *Pneumocystis* were identified using immunofluorescence stain (MeriFluo Kit Biosciences, Cincinnati, Ohio) in the 128 infants. Each sample was analyzed separately and blinded to nPCR results. The 3 lobes per infant were analyzed in duplicate for each lobe.

### Additional Microscopy Methods

The first 36 of the 128 infant samples were additionally studied using Gomori-Grocott methenamine silver and Rapid Giemsa (Diff-Quick) staining of lung section imprints. For either microscopy technique, infants were considered “positive” when typical *Pneumocystis* forms were identified and agreed on by 2 observers (R.B. and C.P. or S.L.V.) in 1 or more lobes and “negative” if the 3 lobes contained no *Pneumocystis*. Interpretation was performed blinded to the results obtained using other techniques. Microscopy reading took up to 45 minutes per patient.

### Samples for *P. jirovecii* and MUC5AC Quantifications

Additional lung samples (1 g) were obtained from 59 infants comprising all 20 *Pneumocystis*-negative infants older than 28 days, and 39 *Pneumocystis*-positive infants of closest possible age. Samples were flash-frozen, pulverized in liquid nitrogen using a mortar and pestle, homogenized, and frozen until quantitative PCR (qPCR) and Western blot analysis.

### *Pneumocystis jirovecii* Quantification

DNA was extracted from a 0.4-g aliquot. The multicopy *msg* gene was selected as target using primers PC MSG Forward (5′-CAA AAA TAA CAY TSA CAT CAA CRA GG-3′) and PC MSG Reverse (5′-AAA TCA TGA ACG AAA TAA CCA TTG C-3′) generating a fragment of 156 bp [23] that was cloned in pGEM-T Easy vector (Promega), and used for generating a calibration curve (range of 1 × 10^1^ to 1 × 10^6^ copies/μL). Amplified product was detected using SYBR Green I (Quantace, Bioscan). Quantitative PCR was done in triplicate using the LightCycler 2.0 (Roche) with preincubation period of 10 minutes at 95°C and 46 cycles of 10 seconds at 95°C, 10 seconds at 53°C, and 20 seconds at 72°C each, ending with 7 minutes at 72°C. Each run included negative (ultrapure H_2_O) and positive (DNA from a patient with *Pneumocystis* pneumonia) controls and 3 different plasmid standards used in the calibration curve. The specificity of amplified products was verified by melting-curve analysis. Human β-globin gene was used as internal control and for normalization of results as described [5, 24].

### Mucin Determinations

Each aliquot (0.6 g) and a gastric tissue sample (control) were disrupted using a Tissue Tearor (Biospec) in chilled RIPA-modified lysis buffer. Total protein was quantified in supernatant by Bradford (Bio-Rad). Thirty-microgram aliquots were subject to sodium dodecyl sulfate polyacrylamide gel electrophoresis (4% stacking and 8% resolving Tris-Glycine gels). Proteins were transferred to polyvinylidene difluoride membranes and blocked. Mouse anti-MUC5AC immunoglobulin G (IgG) antibody (1:500, 45M1, SCBT) and goat antimouse IgG horseradish peroxidase (HRP)–conjugated antibody (1:2000, SCBT) were used for MUC5AC detection. Membranes were stripped, blocked, and reprobed using standard antiactin antibodies (goat antiactin IgG, 1:2000, SCBT and donkey antigoat IgG HRP, 1:3000, SCBT). Enhanced chemiluminescence reagent was used for membrane development (Pierce ECL WB Substrate, Thermo Scientific). Films were analyzed with Image J software (National Institutes of Health).

### Statistical Analysis

GraphPad Prism 5 software (San Diego, California) was used to compare prevalence of *Pneumocystis* in explained vs unexplained deaths using χ^2^ with Yates’ correction, *Pneumocystis* (MSG copies) at sequential age intervals using analysis of variance, MUC5AC expression according to *Pneumocystis* presence using unpaired *t* test with Welch's correction, and to analyze the correlation between expression of MUC5AC and *Pneumocystis* MSG copies using Pearson test. A *P* value of <.05 was considered significant.

## RESULTS

### Infants and Lung Sample Characteristics

A total of 669 infants (aged 3 days to 12 months) underwent a legally required autopsy at Servicio Médico Legal during the enrollment period. M.G. conducted 134 infant autopsies that fulfilled entry criteria and in which the right lung was submitted for analysis. Six newborn infants (mean age, 14.8 days; median, 17 days; range, 2–22 days) were excluded because of recent hospitalization, and 128 infants with a median age of 2 months 29 days (mean, 3 months 11 days [range, 7 days to 11 months 27 days]), 70 (54.7%) male, were considered for this study. Infants were assigned to specific diagnostic categories after autopsy completion (Table 1). Complete right lungs were obtained in 111 infants, 2 lobes in 3 and, 1 lobe in 14, respectively.

**Table 1.** Detection of *Pneumocystis* by Nested Polymerase Chain Reaction in Homogenized Lung-Tissue Autopsy Specimens of Different Pulmonary Lobes from 128 Infants Dying Suddenly and Unexpectedly in the Community

		*Pneumocystis* DNA		
		Contribution to Diagnosis—Any Lobe^c^		
Autopsy Result	No.^a^	RUL	RML or RLL	Total
Unexplained death	85	61	10	71 (83.5%)
Unexplained death with mild autopsy findings	28	18	6	24 (85.7%)
Nonspecific lung inflammation	15			
Congenital malformation (compatible with life)	4			
Metabolic defect (hypoglycemia)	1			
Signs of infection (mild and outside the lung)	8			
Explained death	15	6	4	10 (66.7%)
Bronchopneumonia	4			
Congenital malformation (cardiac or brain)	2			
Traumatic death	2			
Asphyxia (immersion or food)	2			
Systemic signs of infection (DIVC, meningitis, other)	5			
Total	128	85 (80.9%)^b^	20 (19.1%)^b^	105 (82.0%)

Abbreviations: DIVC, disseminated intravascular coagulopathy; RLL, right lower lobe; RML, right middle lobe; RUL, right upper lobe.

^a^ Age: mean, 3 mo 11 d; median, 2 mo 29 d; range, 7 d to 11 mo 27 d.

^b^ Percentage relative to the 105 *Pneumocystis* DNA–positive infants to indicate that 80.9% of positives was detected by analyzing the RUL and 19.1% additional positives by analyzing the RML or RLL specimens. For the purpose of this study, infants were considered to be negative for *Pneumocystis* DNA after analysis of 2 samples in each lobe.

^c^ Prevalence of *Pneumocystis* DNA among unexplained vs explained deaths, *P* = .28.

### Sensitivity of Diagnostic Techniques

**Figure 1.**
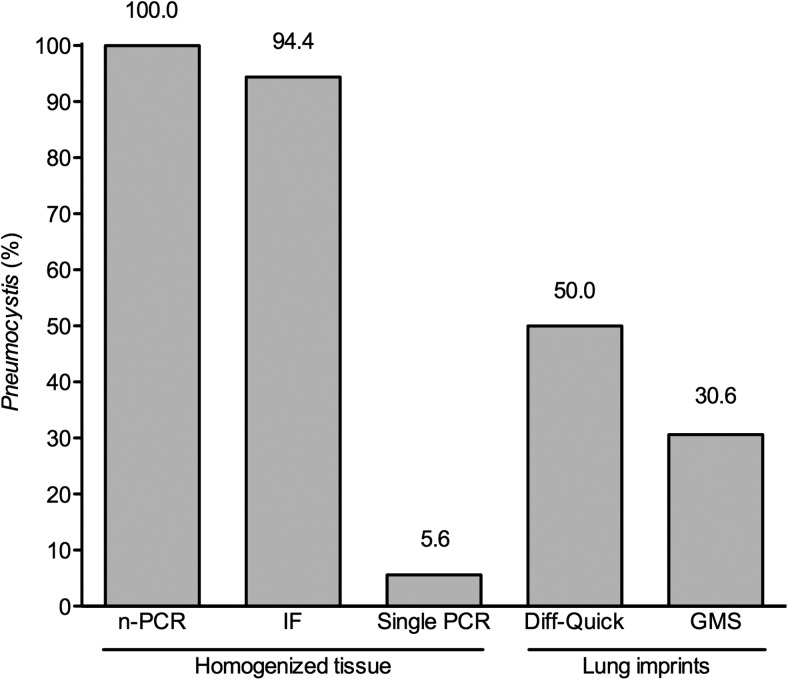
Diagnosis of *Pneumocystis* in infant biopsy specimens requires sensitive techniques applied to homogenized tissue: Percentage of *Pneumocystis* detection as relative to nested polymerase chain reaction (n-PCR), of immunofluorescence microscopy (IF), and single-round PCR in homogenized lung tissue specimens of 36 infants. Results of microscopy readings using rapid Giemsa (Diff-Quick) and Gomori-Grocott methenamine silver (GMS) stains in imprints of cruent-cut-surface lung tissue adjacent to the sections analyzed by n-PCR and IF are also presented. Abbreviations: IF, immunofluorescence microscopy; GMS, Gomori-Grocott methenamine silver; n-PCR, nested polymerase chain reaction; PCR, polymerase chain reaction.

**Figure 2.**
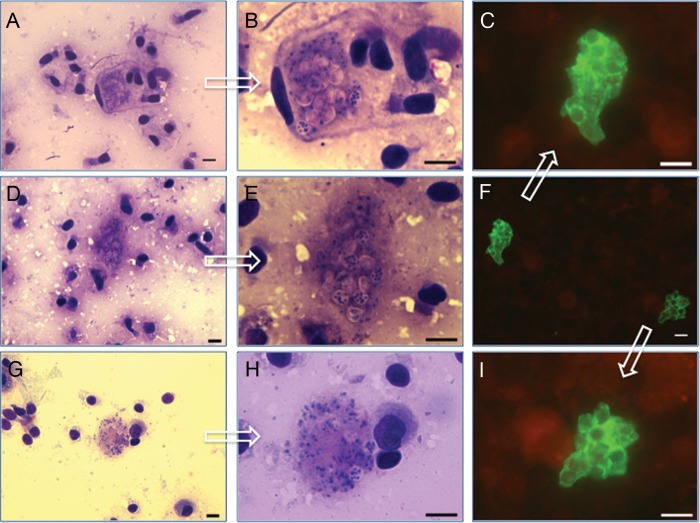
Detection of this highly focal *Pneumocystis* infection by microscopy examination in homogenized preparations or imprints from lung tissue specimens. *Pneumocystis* forms as visualized by microscopy using immunofluorescence stain in aliquots of homogenized lung biopsy specimens (*F* = ×400; *C* and *I* = ×1000), or by rapid Giemsa stain (Diff-Quick) in imprints from fresh lung infant autopsy specimen sections (*A*, *D*, and *G* = ×400; *B*, *F*, and *H* = ×1000). Arrows on each ×400 picture point to their ×1000 magnifications. Bar = 10μ.

*Pneumocystis jirovecii* DNA was detected by nPCR in the first 36 infants studied; 34 (94.4%) of them tested positive by immunofluorescence microscopy, and 2 (5.6%) by single PCR in the same homogenized tissue aliquot. Diff-Quick and Gomori-Grocott methenamine silver stains detected *Pneumocystis* trophic forms in 18 (50.0%) and cyst forms in 11 (30.6%), of lung tissue imprints (Figures 1 and 2).

### DNA Amplification

**Figure 3.**
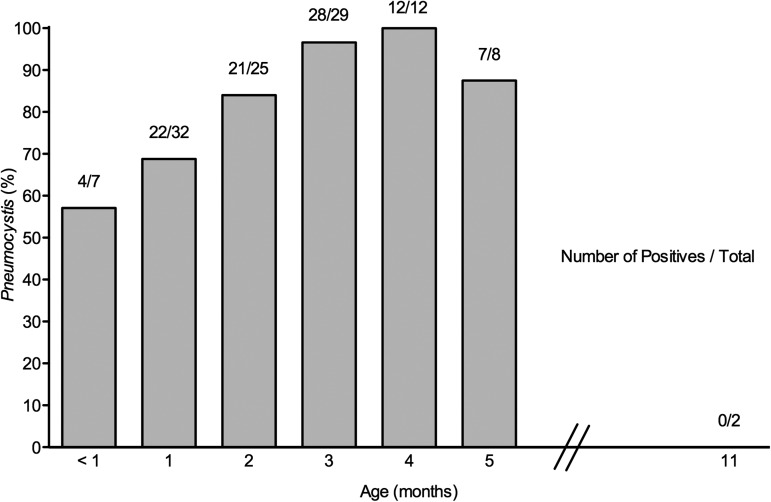
*Pneumocystis jirovecii* infection in autopsied infant lungs peaks at 3–5 months. Lung autopsy specimens from 128 infants dying in the community were analyzed for *P. jirovecii* using nested polymerase chain reaction (nPCR) and immunofluorescence microscopy (IF). *P. jirovecii* DNA was detected in 105 (82.0%), and *Pneumocystis* forms were confirmed by IF in 99 (94.2%) of those found positive for *P. jirovecii* DNA by nPCR and in 0 of 23 infants who tested negative. Each bar represents a minimum of 5 infants. *Pneumocystis* was additionally detected in 4 of 4, 2 of 2, 2 of 3, 2 of 3, 1 of 1, and 0 of 2 infants dying at 6, 7, 8, 9, 10, and 11 months of age, respectively.

Nested PCR detected *P. jirovecii* DNA in 105 (82.0%) of the 128 infants: 60 (85.7%) of 70 male and 45 (77.6%) of 58 female infants. *Pneumocystic jirovecii* DNA was detected in 88 (79.3%) of 111 infants having their 3 lobes analyzed; of them, 35 (39.8%), 21 (23.9%), and 32 (36.3%) had detectable *P. jirovecii* DNA in 3, 2, or 1 lobes, respectively. The first analysis detected 80 (94%) of the 85 infants whose right upper lobe (RUL) was *P. jirovecii* DNA positive (Table 1). *Pneumocystis jirovecii* DNA was detected in 4 of 7 infants < 1 month of age (Figure 3). All amplification reactions of controls for contamination of DNA extraction and purification were negative.

### Microscopy Analyses

Lung homogenate specimens from the 128 infants were analyzed by immunofluorescence microscopy in addition to nPCR, and cystic plus smaller trophic *Pneumocystis* forms were detected in 99 (94.3%) of 105 infants testing positive by nPCR. Immunofluorescence was negative in all 23 infants who were *Pneumocystis* DNA negative by nPCR (Table 1; Figure 2).

### *Pneumocystis* Quantification

**Figure 4.**
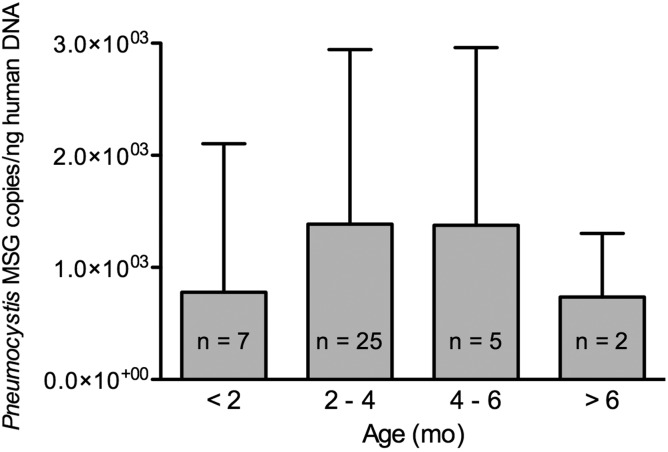
*Pneumocystis* organisms burden increases up to 3–5 months of infant age and declines thereafter. Age progression of *Pneumocystis* organisms load in autopsy lung samples from 39 infants dying suddenly in the community is shown. *Pneumocystis* MSG quantitative polymerase chain reaction results were normalized to nanograms of human β-globin DNA for comparisons and expressed as the normalized mean of *Pneumocystis* MSG copies ± SD.

*Pneumocystis* normalized counts (MSG copies per nanogram of human DNA) were higher between 2 and 5 months and declined thereafter (*P* = .7630) (Figure 4).

### MUC5AC Determinations

**Figure 5.**
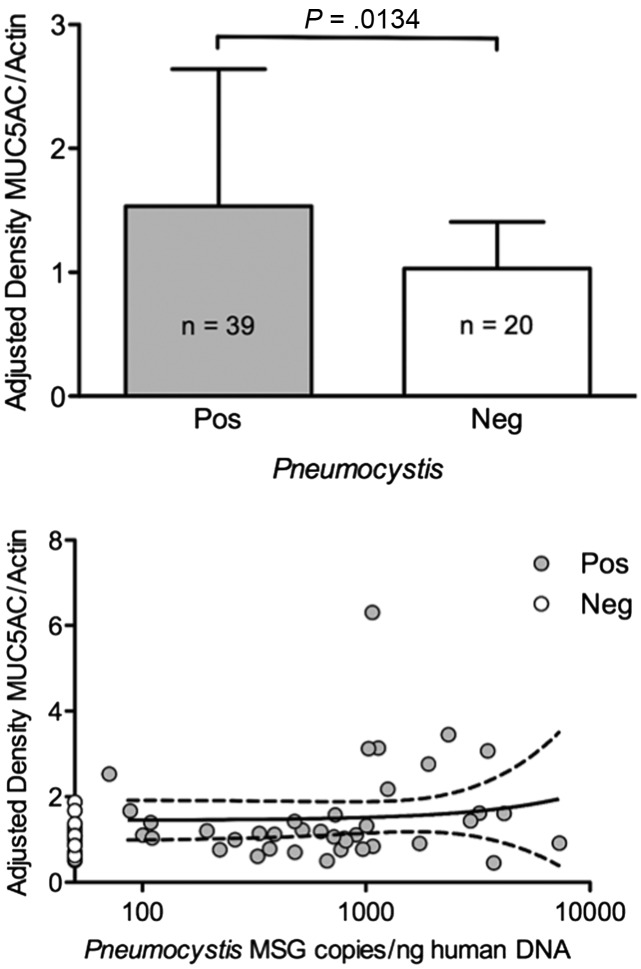
Mucus (MUC5AC) expression is increased by *Pneumocystis* presence and not influenced by organism load. Top: MUC5AC protein expression according to *Pneumocystis* status in lung tissue specimens from 39 *P. jirovecii*–positive and 20 *P. jirovecii*–negative infants (mean ± SD). Bottom: Correlation between normalized MUC5AC protein expression and normalized quantification values of *P. jirovecii* MSG in the same lung sample specimen for each infant (Pearson *r* = 0.0908, *P* = .5822). MUC5AC level values were normalized by human actin protein expression, and *Pneumocystis* MSG determinations by human β-globin levels (mean ± SD). Abbreviation: MUC5AC, mucus.

Normalized levels of MUC5AC were significantly increased (*P* = .0134) in association with the presence of *Pneumocystis* (Figure 5). This increase was consistent at all age intervals (data not shown), and independent of *Pneumocystis* burden (Pearson *r* = 0.0908; *P* = .5822). MUC5AC determination values were normalized by human actin protein expression, and *Pneumocystis* MSG determinations by human β-globin levels (mean ± SD).

## DISCUSSION

This study confirms *Pneumocystis* as the most prevalent microorganism in autopsied infant lungs identified to date, and that *Pneumocystis* presence associates to increased mucus (MUC5AC) expression, suggesting that it increases the mucociliary clearance workload and upregulates innate immune responses in the airway epithelium [19–21].

*Pneumocystis* cells and *P. jirovecii*–specific DNA were identified in the lungs of nearly all infants in this study using immunofluorescence microscopy and nPCR, respectively. This high prevalence is consistent with previous evidence that *Pneumocystis* is common in infant lungs [9, 10] including a study documenting *Pneumocystis* DNA by nPCR in all of 58 infants of undisclosed age [25]. Furthermore, the structural forms of the fungus were all recognized using Giemsa and GMS stains, suggesting active replication [26].

The comprehensive diagnostic approach utilized in this study, including examination of up to 6 fresh homogenized tissue samples per infant, increased the sensitivity of detection and underlines the focal distribution of *Pneumocystis* in the nonimmunocompromised host [8, 9]. This approach detects smaller burdens of *Pneumocystis* than present in immunocompromised patients with *Pneumocystis* pneumonia, where the fungus is readily diagnosable by microscopy or single-round PCR. *Pneumocystis* burden in these infant lungs, although mild, was greater than in immunocompetent adults where diagnosis additionally requires of tissue-concentration techniques [22].

In addition, results show that the age peak with approximately 90% of infants having detectable *Pneumocystis,* and the higher normalized burden of organisms, coincide at 2–5 months. This age predominance was suggested in previous studies [9–11] and matches the age of onset of severe *Pneumocystis* pneumonia in immunosuppressed or debilitated infants prior to anti-*Pneumocystis* prophylaxis [27, 28]. Importantly, young age is by itself a risk factor for *Pneumocystis* severity exemplified by the worse prognosis of HIV-related *Pneumocystis* pneumonia in infants whose mortality is 60% vs 10% in adults [27, 28].

This study also documents that *Pneumocystis* is associated with increased mucus production. Mucus is a gel composed by water (97%) and solids including mucins (3%) [19, 20]. MUC5AC, the gel-forming mucin used as a marker of mucus in this study, is the predominant solid component of mucus in infant airways [29]. Increased normalized levels of MUC5AC have been similarly documented in association with many other well-recognized, less prevalent airway offenders like respiratory viruses, bacteria, acetyl choline, cytokines, prostaglandins, lipopolysaccharides, nitric oxide, and other potential activators of nonspecific airway signaling pathways as the ErbB receptor epidermal growth factor receptor (EGFR) [20, 21]. Additional airway offenders were not studied. MU5AC was consistently increased in *Pneumocystis*-positive infants at all age intervals, suggesting that *Pneumocystis* predisposes the host to augmented mucus responses during this age period [19–21], and was unaffected by *Pneumocystis* burden in agreement with the concept that pathogenesis for *Pneumocystis* is mostly host dependent [6, 27, 30].

Pathogenically, mucins are heavily glycosylated proteins stored in packaging intracellular granules [31]. Their release in response to airway insults is followed by immediate mucin hydration leading to several hundred-fold intraluminal volume increase in milliseconds [19–21, 31]. This mechanism could represent a risk for narrow, developing infant airways because minor height volume changes in the airway surface liquid can lead to small airway closure in times as short as a breathing cycle [32]. The clinical outcome of increased mucus depends on several factors affecting clearance including airway surface tension, geometry, size, and effective cough [32, 33]. Infants have airways of small diameter, with greater elasticity and compliance, fewer collateral airway channels, and a reduced functional residual capacity, compared with older children or adults [34]. In addition, mucins in infants are more acidic that may reflect greater viscosity [29, 34]. The presence of *Pneumocystis* could therefore favor airway collapse suggested as a mechanism in current hypotheses for SUID [35, 36]. This may occur with few clinical manifestations until most of the peripheral airways are occluded [21]. Airway collapse would be challenging to diagnose at autopsy as it may immediately resolve with postmortem airway relaxation. In addition, gravitational orientation of the lungs and the release of transpulmonary pressure upon opening the thorax may mobilize airway secretions and further decrease autopsy evidence.

*Pneumocystis* is common in the general population at any age. Therefore, *Pneumocystis*-associated mucus increase may also be relevant for chronic respiratory diseases such as chronic obstructive pulmonary disease and cystic fibrosis in which the coexistence of mucus excess and *Pneumocystis* is described [19, 37, 38].

Other pathways increase mucin in addition to the EGFR in the ErbB family of receptors, and include tumor necrosis factor α, STAT6, interleukin 1β, interleukin 13, and NF-κB and may be activated by *Pneumocystis* [16, 17, 30, 39]. In addition, *Pneumocystis* may induce collateral sensitization to a nonspecific antigen in immunocompetent mice, increasing the number of CD45^+^CD11c^+^ antigen-presenting cells that explain an hyper-reactive response upon a later challenge [16]. An airway hyperreactive response can explain airway collapse as documented in sensitized mice [40]. This type of response may be relevant to SUID and infant bronchiolitis whose peak incidences coincide with the age peak of *Pneumocystis* [12, 13, 35, 41].

This autopsy study was conducted in sudden unexpected infant deaths. This is the most frequent form of death in apparently healthy, nonimmunocompromised infants [12]. *Pneumocystis* prevalence was not different in infants with unexplained vs explained deaths in this study, in agreement with a previous study documenting a similar incidence of *Pneumocystis* in infants with unexplained deaths vs in those of similar age dying of accidental causes, confirming that *Pneumocystis* is not sufficient to cause SUID [11]. The high prevalence of *Pneumocystis* in SUID, however, raises the possibility that *Pneumocystis* may be a “necessary but not sufficient” cause of SUID as coadjuvant to diverse nonspecific triggers acting on top of *Pneumocystis*.

Pathology reports in this study showed that inflammation was absent or too mild to explain infant deaths through inflammatory mechanisms, as in previous autopsy series [9]. Autopsy signs of a mild respiratory infection that per se does not explain death are present in approximately half of SUID cases [12]. The lack of evident inflammation in these infants can be explained by death occurring before inflammation develops, or by other reasons including focality of the infection [14]. Animal models demonstrate that the sequence of events leading to lymphocytic response is well demarcated [14, 42], and delayed during low-burden infections such as this one, until *Pneumocystis* multiplies and is able to induce the transient inflammation that eliminates the pathogen in the immunocompetent host [30].

Airway collapse may be favored by increased mucus and could explain death in a proportion of these infants [35, 36], suggesting that prevention of *Pneumocystis-*associated mucus increase until the airway is more developed could reduce vulnerability to SUID and, eventually, to bronchiolitis.

*Pneumocystis* is the most prevalent microorganism in the lungs of small infants. *Pneumocystis*-associated mucus increase may also be relevant to older children or adults with respiratory conditions associated with *Pneumocystis* and increased mucus.
